# Recent HIV Infection Among Men Who Have Sex with Men, Transgender Women, and Genderqueer Individuals with Newly Diagnosed HIV Infection in Zimbabwe: Results from a Respondent-Driven Sampling Survey

**DOI:** 10.1089/aid.2021.0216

**Published:** 2022-11-03

**Authors:** Lauren Elizabeth Parmley, Tiffany G. Harris, Avi J. Hakim, Godfrey Musuka, Innocent Chingombe, Owen Mugurungi, Brian Moyo, Munyaradzi Mapingure, Perpetua Gozhora, Chesterfield Samba, John H. Rogers

**Affiliations:** ^1^ ICAP at Columbia University, New York, New York, USA.; ^2^ Department of Epidemiology, Mailman School of Public Health, Columbia University, New York, New York, USA.; ^3^ Division of Global HIV and TB, U.S. Centers for Disease Control and Prevention, Atlanta, Georgia, USA.; ^4^ ICAP at Columbia University, Harare, Zimbabwe.; ^5^ Ministry of Health and Child Care, Harare, Zimbabwe.; ^6^ GALZ, Harare, Zimbabwe.; ^7^ Division of Global HIV and TB, U.S. Centers for Disease Control and Prevention, Harare, Zimbabwe.

**Keywords:** men who have sex with men, transgender people, recent HIV infection, Africa

## Abstract

In Africa, rapid testing for recent HIV infection (RTRI) is being scaled up; however, use of the recent infection testing algorithm (RITA), which uses viral load (VL) to confirm RTRI-recent infections, is not a widespread practice. We present results of recently acquired HIV infections among men who have sex with men (MSM), transgender women, and genderqueer (TGW/GQ) individuals with newly diagnosed HIV infection in Zimbabwe as per the national approach (RTRI) and applying a RITA. In 2019, 1,538 MSM and TGW/GQ in Harare and Bulawayo, Zimbabwe were recruited to participate in a biobehavioral survey using respondent-driven sampling. Consenting participants received HIV testing and all HIV-positive specimens were tested with the RTRI Asanté HIV-1 Rapid Recency Assay, and for VL and CD4 count. RTRI-recent participants with unsuppressed VL (≥1,000 copies/mL) were classified as RITA-recent. Descriptive statistics were used to summarize results among RTRI-recent and RITA-recent participants. Among those tested for HIV (1,511/1,538), 22.5% (340/1,511) tested positive and of those, 55.0% (187/340) self-reported an HIV-negative or unknown status. Among these, 8.6% (16/187) were classified as RTRI-recent and 91.4% (171/187) were classified as RTRI-long term. After accounting for VL, RITA-recency was 1.1% (2/187). Two of 16 (12.5%) RTRI-recent infections were RITA-recent. VL among RITA-recent cases were 9,052 copies/mL and 40,694 copies/mL and both had CD4 counts <500. Data highlight misclassification of recent infections among MSM and TGW/GQ with newly diagnosed HIV infection using RTRI. With the incorporation of VL, >85% of RTRI-recent cases were reclassified as RITA-long term. True characterization of recent infections may not be possible without VL testing, which remains challenging in resource-limited settings.

## Introduction

Antibody-based tests that can help distinguish recent from long-term HIV infection have been used since the mid-1990s to estimate population-level HIV incidence and to evaluate the impact of large-scale HIV interventions in preventing HIV infection.^1,2^ Point-of-care rapid testing for recent HIV infection (RTRI), which aims to detect HIV infection acquired within 12 months, has recently become available and is being scaled up under the U.S. President's Emergency Plan for AIDS Relief (PEPFAR) to describe shifting dynamics in the HIV epidemic and to interrupt active HIV transmission networks.^3–6^ To minimize the proportion false recent (PFR), viral load (VL) testing is used to confirm a recent infection as part of a recent infection testing algorithm (RITA),^7–10^ but is not practiced in all PEPFAR programs in Africa, where the PFR can be high,^11,12^ due to limits in the availability of VL testing.

Since 2019, Zimbabwe has rolled out HIV recency testing nationally as part of HIV case-based surveillance but does not incorporate VL (RITA) due to resource limitations. The objective of this analysis was to present results of recently acquired HIV infection as per the national approach (RTRI) and applying a RITA among 187 men who have sex with men (MSM), transgender women, and genderqueer (TGW/GQ) participants with newly diagnosed HIV infection enrolled in a biobehavioral survey (BBS) in Zimbabwe, the first BBS among MSM and TGW/GQ in the country and, to our knowledge, the first survey to use the Asanté HIV-1 Rapid Recency Assay (Asante) among key populations (KP) in Southern Africa.

## Materials and Methods

### Data collection

Between March and July 2019, ICAP at Columbia University in collaboration with the Zimbabwe Ministry of Health and Child Care and U.S. Centers for Disease Control and Prevention (CDC) Zimbabwe conducted a cross-sectional BBS with MSM and TGW/GQ individuals in Harare and Bulawayo, Zimbabwe. The survey was conducted in accordance with the World Health Organization's BBS Guidelines for Populations at Risk for HIV^13^ and full details on the methods and primary outcomes have been published.^14,15^ Briefly, individuals were recruited using respondent-driven sampling (RDS), a chain referral approach used to recruit populations for whom no sampling frame exists.^16^ Individuals were eligible to participate if they were assigned male sex at birth; engaged in oral or anal sex with a man in the past 12 months; were 18 years or older; resided in Harare or Bulawayo for at least 1 month; spoke English, Shona, or Ndebele; provided written informed consent; and possessed a valid recruitment coupon (for candidate participants).

Survey staff administered a structured questionnaire to all participants through tablet. All consenting participants were tested for HIV regardless of their self-reported status using an adapted version of the national three-test algorithm (Alere HIV Combo, Chembio HIV1/2 STAT-PAK, INSTI HIV1/2). All HIV-positive specimens were tested for recent infection using Asante, as well as for VL and CD4 count. Individuals who self-reported they were HIV negative or unaware of their status and tested HIV positive as part of the survey were considered to have newly diagnosed HIV infection. Individuals with newly diagnosed HIV infection who tested RTRI-recent with unsuppressed VL (HIV RNA ≥1,000 copies/mL) were classified as RITA-recent. Those with suppressed VL (HIV RNA <1,000 copies/mL) were deemed to be likely already on effective antiretroviral therapy (ART) without disclosure of status (probable long-term infection).

### Data analysis

Data analysis was conducted in SAS (version 9.4; SAS Institute). Primary analyses were restricted to participants with newly diagnosed HIV infection to reflect how the test is used in practice. Bivariate analyses, including chi-square tests with continuity adjustment and Fisher exact tests, were used to summarize demographic and biomarker results by KP (MSM, TGW/GQ). Univariate analyses were used to compare RTRI-recent cases without VL, and RTRI-recent cases confirmed with VL (RITA-recent). A subanalysis among participants who reported a previous HIV diagnosis but were RITA-recent was also explored and results reported. The RDS sample did not reach convergence on key variables, including HIV, and was treated as a convenience sample. Survey data from both cities were combined for analysis due to small numbers.

### Ethical considerations

Ethical and administrative approvals were received from the Columbia University Institutional Review Board and the Medical Research Council of Zimbabwe. The protocol was also reviewed in accordance with the CDC human research protection procedures and was determined to be research, but CDC investigators did not interact with human subjects or have access to identifiable data or specimens for research purposes. All participants provided written informed consent for survey participation and biomarker testing separately. Participants were reimbursed US$5 for participation and an additional US$5 for each successful recruit (three maximum).

## Results

Among individuals screened for eligibility in Harare and Bulawayo, 85.9% (718/836) and 81.3% (820/1,009), respectively, were eligible to participate and, of those, 100.0% in both cities enrolled in the survey resulting in 718 participants in Harare (MSM: 431; TGW/GQ: 287) and 820 in Bulawayo (MSM: 763; TGW/GQ: 57). Almost all participants (Harare: 96.8%; Bulawayo: 99.5%) consented to biomarker testing.

Among those who consented and were tested for HIV (1,511/1,538), 22.5% (340/1,511) tested positive (MSM: 21.1%; TGW/GQ: 27.5%) and of those, 55.0% (187/340) self-reported an unknown or HIV-negative status before testing (MSM: 52.0%; TGW/GQ: 63.0%). While most self-reported newly diagnosed HIV infections were among participants 25–34 years of age (46.0%; 86/187) (Table 1), there were significant differences in age between MSM and TGW/GQ (*p* < .001) with more than half (53.4%; 31/58) of TGW/GQ between 18 and 24 years compared with 14.7% (19/129) of MSM between 18 and 24 years. Among those with self-reported newly diagnosed HIV infection, 8.6% (16/187) were classified as RTRI-recent (MSM: 4.7%; TGW/GQ: 17.2%, *p* = .009) and 91.4% (171/187) were classified as RTRI-long term (MSM: 95.4%; TGW/GQ: 82.8%).

**Table 1.** Demographic and HIV Biomarkers Among Men Who Have Sex With Men, Transgender Women, and Genderqueer Individuals Who Self-Reported Newly Diagnosed HIV Infection, Zimbabwe, 2019

Demographic and HIV biomarkers among MSM, TGW and GQ individuals who self-reported newly diagnosed HIV infection by key population, Harare and Bulawayo, Zimbabwe 2019						
	MSM (*n* = 129)		TGW/GQ (*n* = 58)		Total (*n* = 187)	
	col%	n	col%	n	col%	N
Age						
18–24	14.7	19	53.4	31	26.7	50
25–34	50.4	65	36.2	21	46.0	86
35+	34.9	45	10.3	6	27.3	51
City						
Harare	36.4	47	86.2	50	51.9	97
Bulawayo	63.6	82	13.8	8	48.1	90
Employment status						
Self employed	33.3	43	27.6	16	31.6	59
Full/part-time employment	27.9	36	29.3	17	28.3	53
Unemployed	31.8	41	29.3	17	31.0	58
Other (student/retired)	7.0	9	13.8	8	9.1	17
Marital status						
Single, never married	69.8	90	75.9	44	71.7	134
Married/cohabitating	8.5	11	5.2	3	7.5	14
Separated/divorced	21.7	28	19.0	11	20.9	39
Sexual orientation						
Gay/homosexual	68.2	88	72.4	42	69.5	130
Bisexual	30.2	39	25.9	15	28.9	54
Straight/heterosexual	0.8	1	0	0	0.53	1
Other	0.8	1	1.72	1	1.1	2
RTRI						
Recent infection	4.7	6	17.2	10	8.6	16
Long-term infection	95.3	123	82.8	48	91.4	171
Inconclusive	0	0	0	0	0	0
Invalid	0	0	0	0	0	0
RITA						
Recent infection	0	0	3.4	2	1.1	2
Long-term infection	100	129	96.6	56	98.9	185
CD4 count						
<200	13.2	17	3.4	2	10.2	19
200–350	27.1	35	22.4	13	25.7	48
350–500	28.7	37	36.2	21	31.0	58
>500	31.0	40	37.9	22	33.2	62
Median (IQR)	393 (284–562)		453.5 (343–583)		413 (305–573)	
Viral load result						
Suppressed (<1,000 copies/mL)	51.9	67	41.4	24	48.7	91
Not suppressed (≥1,000 copies/mL)	48.1	62	58.6	34	51.3	96

RTRI inconclusive indicates participant tested HIV positive according to the HIV testing algorithm but tested HIV negative through RTRI. RTRI invalid indicates an inadequate or improper sample was collected, the assay was not performed correctly, or the assay was not functioning properly. RITA-recent indicates participant tested RTRI-recent and had unsuppressed viral load (HIV RNA ≥1,000 copies/mL).

MSM, men who have sex with men; IQR, interquartile range; RITA, recent infection testing algorithm; RTRI, rapid test for recent infection; TGW/GQ, transgender women/genderqueer.

Nearly half (48.7%; 91/187) of those with newly diagnosed HIV infection were virally suppressed (MSM: 51.9%; TGW/GQ: 41.4%) and their median CD4 count was 413 [interquartile range (IQR): 305–573; MSM: 393, IQR: 284–562; TGW/GQ: 453.5, IQR: 343–583]. After accounting for VL, RITA-recency was 1.1% ([2/187] [MSM: 0% (0/129); TGW/GQ: 3.4% (2/58)], *p* > .05) among those with newly diagnosed HIV infection. Of the 16 RTRI-recent infections, 2 (12.5%) were RITA-recent.

Table 2 displays the demographic characteristics of RTRI-recent and RITA-recent cases. The two RITA-recent cases were recruited two waves apart (recruitment waves 10 and 12) within the same recruitment chain. Both identified as TGW/GQ and lived in the same neighborhood in Harare. Only one reported ever testing for HIV and this participant reported receiving an HIV-negative test result 2 months preceding the survey. VL among the RITA-recent cases were 9,052 and 40,694 copies/mL and both had CD4 counts <500 (465 and 493). Both RITA-cases reported condomless receptive anal sex at last sex with their primary male partner.

**Table 2.** Demographic Characteristics Among Rapid Test For Recent Infection- and Recent Infection Testing Algorithm-Recent Men Who Have Sex With Men, Transgender Women, and Genderqueer Individuals Who Self-Reported Newly Diagnosed HIV Infection, Zimbabwe, 2019

Demographic characteristics among RTRI- and RITA-recent MSM, TGW and GQ individuals who self-reported newly diagnosed HIV infection, Harare and Bulawayo, Zimbabwe 2019				
	RTRI-recent (*n* = 16)		RITA-Recent (*n* = 2)	
	%	*n*	%	*n*
City				
Bulawayo	31.3	5	0	0
Harare	68.8	11	100.0	2
Age				
18–24	43.8	7	50.0	1
25–34	37.5	6	50.0	1
35+	18.8	3	0	0
Key population				
MSM	37.5	6	0	0
TGW/GQ	62.5	10	100.0	2

While the recency assay is intended for use in individuals with newly diagnosed HIV infection, given the design of the survey and that all participants were tested for HIV regardless of self-reported HIV status and that all HIV-positive specimens were tested for recency, we also explored RITA recency among those with self-reported diagnosed HIV before the survey (3/153). Of the three RITA-recent cases among previously diagnosed participants, all were MSM living in Bulawayo. Two reported being diagnosed (and initiating ART) within the six months preceding the survey, consistent with the assay's detection of a window period of recent infection estimated to be within 6 months.^17^ The third reported taking ART since 2009. This participant had a VL of 148,244 and a CD4 count of 41, indicative of acquired immunodeficiency syndrome and probable ART failure.

## Discussion

Data presented highlight misclassification of recent infections using RTRI among KP with self-reported newly diagnosed HIV infection, underscoring the importance of VL testing to increase testing accuracy for recent HIV infections. With the incorporation of VL results, >85% of RTRI-recent cases were classified as RITA-long term. This discrepancy in RTRI- and RITA-recent infection is due to high PFR and test performance, which has low positive predictive value of recent infections without VL in a population where incidence is low. Given the high levels of VL suppression among those with newly diagnosed HIV infection, we believe a substantial proportion of participants failed to disclose their HIV and ART status. Reliance on self-reported newly diagnosed HIV infection combined with RTRI only per the current national approach can result in the misclassification of many long-term infections as recent.

Failure to disclose HIV diagnosis and ART is not unique to our survey. A recent global meta-analysis found the prevalence of underreporting known HIV-positive status was 20% (95% confidence interval: 13–26) and was higher among MSM.^18^ This phenomenon was also documented in both rounds of the Zimbabwe Population-based HIV Impact Assessments (ZIMPHIA), nationally representative surveys to measure population-level HIV prevalence and incidence.^19,20^ In the latest ZIMPHIA, nearly a quarter of participants underreported known HIV-positive status and 18.4% who reported a previous diagnosis but no ART use had detectable levels of antiretrovirals (ARVs) in their blood.^20^ Countries implementing recency testing, particularly those implementing RTRI alone, should ensure that pretest counseling entails careful probing for both HIV and ART status, before testing, and surveys implementing the Asante assay may consider ARV and VL testing if resources allow.

High PFR of the Asante assay has been found among patients on effective ART (>50%) and elite controllers (>10%), reinforcing global recommendations to integrate VL testing (RITA) to determine recent infection and estimate incidence.^21^ To our knowledge, our survey is the first to publish PFR among KP specifically with the point-of-care assay. While PFR is context dependent and is unknown in Zimbabwe as validation of the Asante assay for recent infection in the country did not specifically address use of VL, the PFR among KP in this survey was higher than PFR reported elsewhere among the general population,^21^ including among patients on ART, and higher than the PFR of BED-CEIA and LAg-Avidity EIA among KP reported elsewhere.^10^

Globally, HIV incidence among MSM and transgender people have increased by 25% and 5%, respectively, since 2010, and account for 25% of all new HIV infections combined.^22,23^ In this sample, prevalence of recent HIV infection was 1.1% among those with newly diagnosed HIV infection. As PEPFAR programs roll out recency testing, countries unable to fully scale RITA due to resources may consider prioritizing RITA among these populations to inform site- and above-site response more accurately.

While RITA is unavailable in Zimbabwe, RTRI-recency among 297 MSM with newly diagnosed HIV infection who tested for recent HIV infection through Zimbabwe's HIV case surveillance program from 2019 to 2020 was 13.8%,^24^ 1.6 times higher than RTRI-recency in this sample. Compared with BBS participants, patients actively seeking HIV testing services may have higher RTRI-recency due to recent engagement in sexual or injection risk behaviors, which may motivate testing, although given high recency misclassification in this sample, RTRI-recency must be used with caution.

The main limitations of this article are the small number of recent HIV infections, which did not allow for statistical analysis. The Asante assay has not been extensively validated for different HIV-1 subtypes but new evidence suggests recent infection results may vary based on subtype; in a recent assay performance, the Asante assay underreported recent infections among individuals with subtype C, the dominant HIV-1 subtype in Zimbabwe and much of Southern Africa, compared with individuals with subtype D, more commonly found in East Africa.^25^

An additional limitation of the survey was that we did not test for the presence of ARVs which, in combination with VL,^7^ would help minimize PFR. Moreover, as this RDS sample is unweighted, results may not be generalizable to all MSM and TGW/GQ in the survey towns. However, given limited data on use of recency testing among KP and applications of the Asante assay, we believe these data are of value to HIV programs and surveillance efforts implementing recency testing. While point-of-care testing for recent HIV infection offers opportunities to monitor epidemic trends, to accurately characterize recent infections among these groups, VL testing is needed.

## Conclusions

In this sample, 8.6% of MSM and TGW/GQ with newly diagnosed HIV infection had a recent infection according to RTRI, but confirmatory testing using VL (RITA) reclassified most RTRI-recent misclassified infections, resulting in a 1.1% recent infection prevalence among those with newly diagnosed HIV infection. These findings suggest that true characterization of recent HIV infections may not be possible without use of VL testing and HIV programs should consider including it as part of HIV recency testing if resources allow.
